# A193 PERCEIVED BARRIERS TO GLUTEN-FREE FOOD ACCESS ON-CAMPUS EXPERIENCED BY STUDENTS FROM DIFFERENT CANADIAN UNIVERSITIES AND COLLEGES

**DOI:** 10.1093/jcag/gwab049.192

**Published:** 2022-02-21

**Authors:** J Mistry, M Khaouli, D Weiten, S Case, D Gidrewicz, J Turner, D Duerksen, M I Pinto-Sanchez

**Affiliations:** 1 McMaster University, Hamilton, ON, Canada; 2 Medicine, McMaster University, Hamilton, ON, Canada; 3 University of Manitoba, Winnipeg, MB, Canada; 4 Stollery Children’s Hospital, Edmonton, AB, Canada; 5 University of Calgary, Calgary, AB, Canada; 6 University of Saskatchewan, Saskatoon, SK, Canada

## Abstract

**Background:**

Students with gluten-related disorders (GRD), a spectrum of conditions including celiac disease (CeD) and non-celiac wheat sensitivity (NCWS), often experience challenges when accessing gluten-free (GF) foods.

**Aims:**

To identify barriers perceived by students with GRD to access GF products on-campus of universities and colleges across Canada.

**Methods:**

We conducted a cross sectional survey using the RedCap platform and distributed it to the Canadian Celiac Association community. We included students who reported adopting a GFD for various reasons including CeD and other GRD. We collected data on adherence to the GFD using a validated questionnaire (CDAT), presence of perceived barriers to follow a GFD while dining on campus, persistent symptoms, and altered quality of life. Continuous data are expressed as median (IQR), and categorical data as proportions of patients. Mann-Whitney U and Chi2 with Fisher correction were used to assess differences between groups.

**Results:**

Seventy nine students responded to the survey (5% male and median age = 25 yrs) and 78 had complete data for analysis. Of the 78 students, 52 (66.6%) reported a diagnosis of CeD, while 26 were adopting a GFD for other reasons (non-CeD). The majority were enrolled in university programs (72/78) and 18% were living on-campus. Almost 90% reported difficulties maintaining a GFD while dining on-campus. Similar proportion of CeD and non-CeD reported eating gluten accidentally (75% vs 80%), while 15% reported eating gluten intentionally on-campus at least a few times per week. This was observed more frequently in non-CeD compared with students with CeD (61% vs 17%; p=0.04). Barriers identified in CeD versus non-CeD groups were related to a reduced GF-food variety (48% vs 69%), lack of availability of GF food (21% vs 46%) and increased cost (46% vs 81%) compared with gluten-containing counterparts. The majority of participants were concerned whether the food available on-campus was truly GF (80% vs 54%) as they reported foods not properly labelled. The majority of participants considered their overall health (79%) and quality of life (65%) was fair to terrible while dining on campus. During the pandemic, 76% of them perceived that it was easier to stick to a GF diet.

**Conclusions:**

Students from various universities and colleges across Canada experience barriers to access GF food on-campus. This has a significant impact on their overall health and quality of life. Proper food labeling, GF certification and improving the variety of GF food on-campus are options for improvement.

**Funding Agencies:**

None

